# Ventricular volumetry in relation to clinical response and overdrainage after shunt surgery in idiopathic normal-pressure hydrocephalus: a three-year prospective study

**DOI:** 10.1186/s12987-026-00835-0

**Published:** 2026-06-30

**Authors:** Rafael T. Holmgren, Andreas Eleftheriou, Johanna Rydja, Fredrik Lundin, Peter Zsigmond, Charalampos Georgiopoulos

**Affiliations:** 1 Department of Neurosurgery, and Department of Biomedical and Clinical Sciences, Linköping University Hospital, Linköping, 581 85 Sweden; 2 Department of Neurology, and Department of Biomedical and Clinical Sciences, Linköping University, Linköping, Sweden; 3 Department of Health, Medicine and Caring Sciences, Unit of Physiotherapy, Linköping University, Linköping, Sweden; 4 Clinical Department of Activity and Health in Linköping, Linköping, Region Östergötland Sweden; 5 Diagnostic Radiology, Department of Clinical Sciences, Medical Faculty, Lund University, Lund, Sweden; 6 Center for Medical Image Science and Visualization (CMIV), Linköping University, Linköping, Sweden

## Abstract

**Background:**

Idiopathic normal pressure hydrocephalus (iNPH) is treated with shunt surgery, but long-term monitoring of shunt function and complications remains challenging. Traditional radiological measures such as the Evans index lack sensitivity for detecting subtle longitudinal ventricular changes. Ventricular volumetry may provide a more precise method, but prospective long-term data are scarce.

**Objective:**

To evaluate ventricular volumetry in relation to clinical outcome, overdrainage, and valve adjustments during a structured three-year follow-up after shunting for iNPH.

**Methods:**

Fifty patients who fulfilled the diagnostic criteria for iNPH and who underwent shunt surgery with an adjustable Codman Certas Plus® valve without an antisiphoning device (ASD) were enrolled in this prospective single-center study. Clinical outcomes were assessed with the Hellstrom iNPH scale and Mini-Mental State Examination (MMSE) at baseline and at 3, 12, and 36 months. Ventricular volumes were measured via quantitative magnetic resonance imaging (qMRI) and automated segmentation. Overdrainage was defined clinically and/or radiologically.

**Results:**

Forty-two patients completed the 3-month follow-up, 86% of whom were classified as clinical responders. Gait improvement was sustained at 12 months, whereas cognitive gains were transient. The ventricular volume decreased significantly from baseline by a mean of 20% at 3 months (*p* < 0.001), with a further decreasing trend up to 36 months. The responders demonstrated greater relative ventricular reduction (21%) than the nonresponders did (10%) at 3 months, while ventricular reduction was equal at later timepoints. Overdrainage symptoms and events occurred in 12 out of 50 patients, including 7% with subdural collections. Patients with overdrainage symptoms showed markedly greater reductions in ventricular volume (35%) than asymptomatic patients did (16%; <0.001). Valve adjustments resulted in detectable volumetric changes, supporting volumetry as a noninvasive marker of shunt function.

**Conclusion:**

Ventricular volumetry is a sensitive tool for longitudinal follow-up after shunting in iNPH patients. A reduction of ventricular volume of approximately 20% is associated with clinical improvement, whereas a reduction exceeding 30% increases the risk of overdrainage symptoms. Routine baseline volumetry, early intensive follow-up and the routine use of ASDs could be used to optimize outcomes and minimize complications.

**Supplementary Information:**

The online version contains supplementary material available at 10.1186/s12987-026-00835-0.

## Introduction

Idiopathic normal pressure hydrocephalus (iNPH) is a complex neurological disorder that primarily affects older adults and is characterized by the clinical triad of gait disturbance, cognitive dysfunction, and urinary incontinence. Initially described by Hakim et al. in 1965 [1], the prevalence of this condition has increased, with estimates of approximately 400 cases per 100,000 individuals [2] or 1.5% among 70-year-olds [3]. INPH is the most common cause of shunting in most high-income countries, and with an aging population, its prevalence is likely to increase.

Following shunt implantation, long-term evaluation of treatment efficacy is essential, as symptom recurrence can occur and may be caused by shunt malfunction, disease progression or comorbidities mimicking iNPH symptoms. Routine follow-up typically includes both clinical assessments and radiological evaluations. Postoperative imaging focuses primarily on identifying complications such as subdural hematomas, hygromas, and improperly positioned ventricular catheters. Conventional linear measurements, such as frontal horn width and the Evans index, are often inadequate for assessing shunt effectiveness in longitudinal monitoring of shunted iNPH patients, as they lack the sensitivity to detect ventricular changes [4, 5]. A typical clinical scenario involves patients who return with recurring iNPH symptoms, necessitating differentiation between shunt failure and disease progression. Since current imaging techniques often fail to rule out shunt dysfunction, patients frequently undergo invasive procedures such as infusion tests or revision surgeries.

Recent advancements in imaging, such as quantitative magnetic resonance imaging (qMRI), provide new opportunities to assess intracranial volumetry [6]. Compared with conventional 2D measures, ventricular volumetry more precisely and sensitively tracks post shunt volume changes and correlates more strongly with clinical improvement than the Evans index does, indicating that it better reflects patient outcomes [5, 7, 8]. However, clear evidence-based thresholds for adequate ventricular reduction to achieve a treatment effect have not been established.

Overdrainage is a common and increasingly acknowledged problem after shunting in iNPH patients, who typically present with postural headaches and ultimately develop subdural hematomas and hygromas [9]. Visualizing overdrainage with conventional radiological methods in chronic hydrocephalus with large ventricles and noncompliant brain parenchyma can be challenging [10]. Ventricles remain large, and linear measures often remain unchanged, even in severely symptomatic patients. To date, no study has systematically quantified ventricular volumetric changes during symptomatic overdrainage.

In a recent placebo-controlled clinical study by Luciano et al., ventricular volumetry revealed a clear volume reduction in patients with patent shunts at 3 months after surgery [11]. Only a few studies have investigated the long-term trajectories of ventricle volume and subarachnoid CSF volume in shunted iNPH patients [12]. In a 2019 prospective study of 54 shunted iNPH patients, Yamada et al. [13] reported ventricular reduction followed by CSF redistribution from the Sylvian fissure, basal cisterns and posterior fossa to the convexity sulci. However, the study had a significant dropout rate and a wide range of follow-up periods. Overall, the natural history of longitudinal ventricular volume reduction after shunting in iNPH remains insufficiently studied.

To our knowledge, no prospective study has comprehensively evaluated long-term ventricular volumetric trajectories and their associations with clinical outcome, overdrainage events, and valve adjustments in a structured manner. Therefore, the present study aimed to characterize these relationships over a three-year period following shunt surgery for iNPH.

## Method

This was a single-center prospective longitudinal cohort clinical trial. Surgical intervention was performed using a commercially available, noninvasively adjustable differential-pressure shunt valve (Codman Certas Plus® shunt system; Integra Life Sciences Corporation, Plainsboro, NJ, USA). The manufacturer was not involved in the design, conduct, or oversight of the trial. There was no financial or other support provided by the company.

### Study population

Patients referred to our hospital for possible iNPH underwent evaluation following international clinical and radiological guidelines [14] and those who fulfilled the diagnostic criteria for iNPH were consecutively offered to participate in the study upon acceptance for shunt surgery between January 2021 and July 2022. The exclusion criteria included a lumbar ICP exceeding 18 mmHg, lumbar CSF biomarker results indicating alternative causes for symptoms, patients with MRI-incompatible implants such as pacemakers, a short life expectancy, severe cognitive impairments deemed unfit for study participation, and the inability to provide informed consent. Patients with secondary normal pressure hydrocephalus were not included. The participants were assessed for comorbidities and anticoagulant use. The sample size for this cohort was based on previous similar studies [7, 13, 15, 16].

### Ethics approval and consent to participate

The study was conducted in accordance with the Declaration of Helsinki and relevant national regulations. The study was approved by the Swedish Ethical Review Authority (Etikprövningsmyndigheten), reference 2020-00719. All participants provided written informed consent prior to inclusion.

### Trial registration

ClinicalTrials.gov, NCT04785560, registered 2021-01-18.

### Shunt surgery and clinical outcome measures

The day before surgery, clinical baseline examinations according to the Hellstrom iNPH scale [17] and Mini-Mental State Examination (MMSE) [18] were performed by a physiotherapist and occupational therapist. The intake of any anticoagulant or antiplatelet medication was discontinued before surgery according to the department’s routine.

All patients underwent surgery with a right-sided Codman Certas Plus® shunt system (Integra Life Sciences Corporation, Plainsboro, NJ, USA) without an antisiphoning device (ASD), as this was the practice at our department at the time of the study. One of three experienced neurosurgeons performed the surgeries.

Patients underwent postoperative MRI 24–48 hours after surgery. The volumetric results from this examination have been described [8]. All patients were discharged with a valve setting of 4 (=110 mm H2O). Anticoagulant use was started again after a minimum of 10 days after surgery, depending on the substance used.

At 3 months, a complete assessment according to the Hellstrom iNPH scale was performed; at 12 and 36 months, only the motor domains of the scale (gait domain including ordinal rating, 10 m steps and 10 m seconds + balance domain) and MMSE score were investigated. Assessments were performed by the same physiotherapist and occupational therapist at all timepoints. Clinical responders to treatment were defined as having improved from baseline by a minimum of 5 points on the full scale at 3 months and 12.5 points on the gait domain at 12 and 36 months, according to the original paper [17]. The time in seconds needed to walk 10 meters was converted into gait velocity (meters/second). To be included and assessed for study data at a specific timepoint, patients needed to have a functional shunt at that time. This was defined as no clinical suspicion of shunt failure and a flawless investigation for shunt failure in nonresponders.

Any valve adjustments performed from postoperative discharge onwards were noted. Any complications were noted, and patients were specifically asked about any ongoing clinical signs or symptoms of overdrainage, such as the presence of postural headache, tinnitus and vertigo with onset after surgery. Patients who reported such signs or symptoms within the week preceding the visit were defined as clinical overdrainers. Valves were adjusted accordingly in suspicion of over- or underdrainage-related symptoms, and patients were followed up by telephone interview one month later. Patients with subdural hematomas/hygromas and intractable overdrainage had their Certas valves adjusted to setting 8 (a k a “virtual off”, >400 mm H₂O resistance)

### Radiological method

**Fig. 1:**
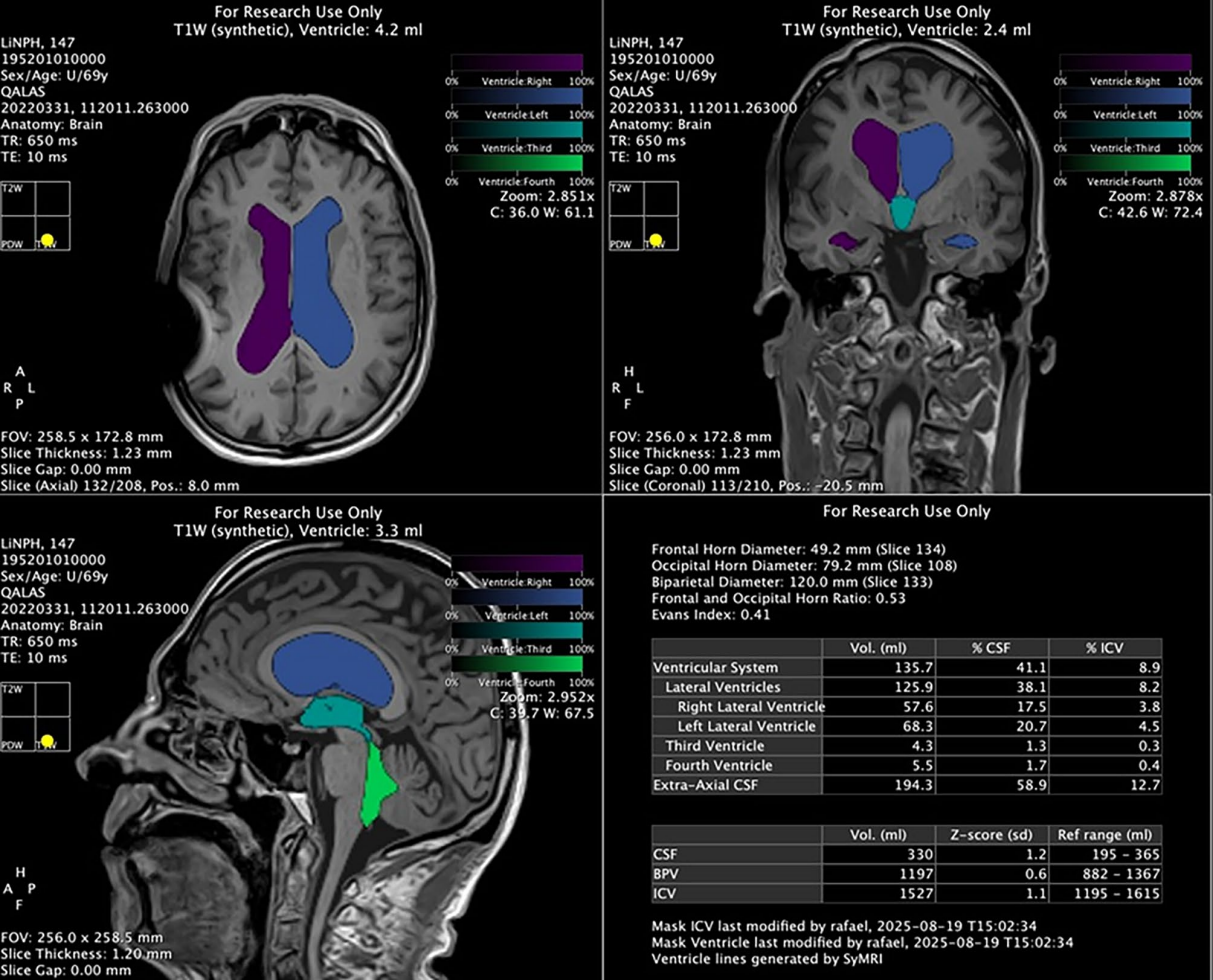
Screenshot of ventricle segmentation and the ventricular volume table from the software SyMRI 15 Research edition v 315.0.19 (SyntheticMR AB®, Sweden)

Pre- and postoperative MRI with qMRI was performed for baseline and follow-up ventricular volumetry. All MRI scans were performed using a 3T Siemens Prisma scanner featuring a 20-channel head coil. The imaging protocol included T1-weighted and T2-weighted sequences, along with a 3D qMRI sequence and 3D-QALAS (3D-quantification using an interleaved look-locker acquisition sequence with a T2 preparation pulse). The 3D-QALAS protocol consists of five parallel, segmented 3D TFE gradient echo acquisitions, interleaved with T2 preparation and inversion pulses, designed to measure T1 and T2 relaxation times and proton density [19]. Using these maps, the software SyMRI 15 Research Edition v 315.0.19 (SyntheticMR AB®, Sweden) automatically determined the partial volume of cerebrospinal fluid (CSF) per voxel. Complete coverage with an isotropic resolution of 1.20 × 1.23×1.23 mm was achieved within a six-minute scanning duration. Volumetric assessments were conducted via 3D-QALAS on synthetic T1- or T2-weighted images produced by SyMRI software. Automatic segmentation of the ventricular system was performed on the basis of the CSF maps via the same software (Fig. 1), which is an updated version of SyMRI v0.45 that has previously been validated and tested for interrater reliability by our group [6]. The total and separated ventricle volumes (lateral, third and fourth) in milliliters were reported. Additionally, the intracranial volume and total intracranial CSF volume were automatically calculated by the software. The imaging artifact caused by the shunt valve on postoperative images was addressed by assuming that the baseline intracranial volume (ICV) remained constant across all subsequent scans and that any observed decrease in ICV at follow-up was entirely due to the signal void generated by the shunt valve artifact. Total intracranial CSF volume and extraventricular CSF volume were therefore adjusted postoperatively using the correction factor ICV(baseline)/ICV(acquisition) in postoperative images. Ventricular volume measurements needed no adjustment, as the extent of the shunt valve artifact did not influence visualization of the ventricular system. The ratios of postoperative volumes normalized against ICV were normalized to the preoperative ICV measurements.

Volumetric image analysis by SyMRI software took less than 30 seconds, corresponding to the time required to process 3D-QALAS images. All the MR images were anonymized and assigned a study identification code for research purposes. Volumetric analyses were performed in a blinded manner to the patients’ clinical outcomes.

### Statistical analysis

The Shapiro‒Wilk test was conducted to evaluate normal distribution. Independent t tests were used for differences between two groups. One-way ANOVA with a Bonferroni adjustment was used for differences between three groups or more. Associations between parametric parameters were examined via Pearson’s correlation analysis, which assumed a linear correlation. Longitudinal differences in volumetric and clinical measures over time were assessed using a repeated measures linear mixed model (rmLMM) with covariance-type compound symmetry. Long-term analyses are affected by attrition; 15 patients did not complete radiological or clinical assessments at all timepoints. Therefore, we report the number of observations at each time point and use rmLMM with robust handling of missing data.

We modelled longitudinal gait velocity using a linear mixed model (LMM) with a patient-level random intercept to account for repeated measures at unevenly spaced visits (baseline, 3, 12, and 36 months). Fixed effects were time (months, continuous), time [2] (to capture the observed curvilinear change), and ventricular volume (ml). LMM was chosen over repeated-measures ANOVA because it (i) accommodates missing data without listwise deletion, (ii) treats time as continuous (0–3–12–36 months), and (iii) permits nonlinear trends via the polynomial term. Model diagnostics indicated approximately normal residuals, homoscedasticity, and no influential outliers.

Power (80%, α = 0.05) was calculated for the paired primary endpoint (ventricular volume change) and to gauge detectable between-group and correlational effects via observed SDs. A 20% mean ventricular reduction from baseline, assuming an SD of 10%, required 5 complete pairs; we enrolled 50 pairs to allow for attrition. Accordingly, the primary endpoint is adequately powered for moderate within-subject change, whereas subgroup analyses have limited power and are considered exploratory. Correlations were powered to detect moderate but not weaker associations.

A *p* value of <0.05 was considered to indicate statistical significance. Statistical analyses were conducted via IBM SPSS Statistics for Windows version 30.

## Results

**Fig. 2:**
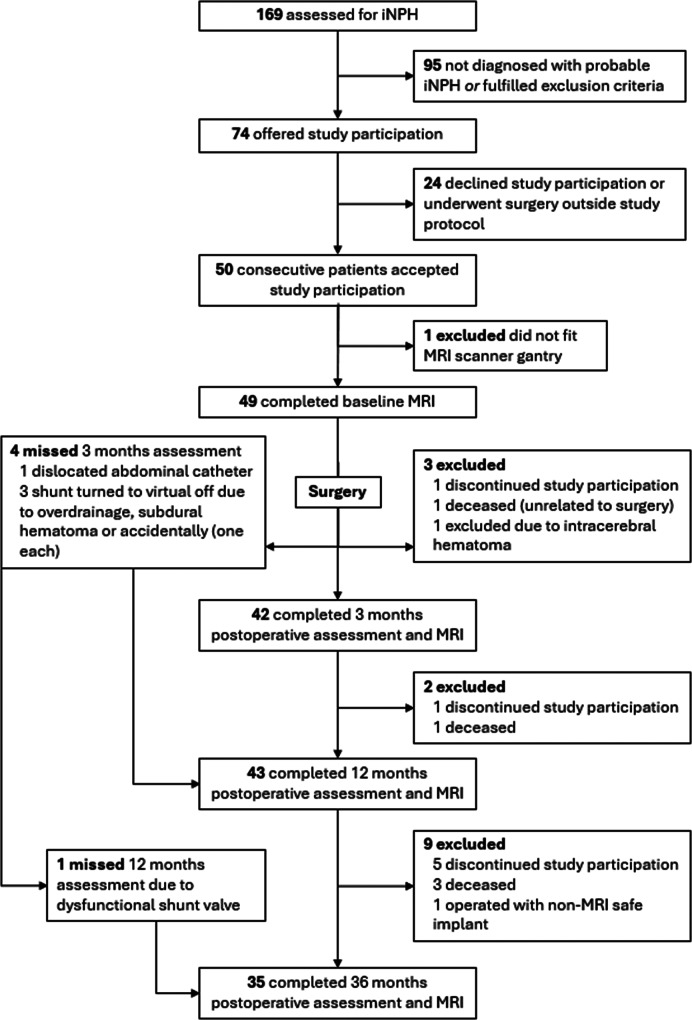
Study workflow, participants and excluded patients

The study workflow and patient discontinuation are shown in Fig. 2. The first 50 patients who agreed to participate were enrolled in the study; 49 completed baseline preoperative imaging, 42 were assessed at 3 months, 43 at 12 months, and 35 at 36 months. Thirty patients completed all three postoperative follow-up visits without interruption. The average age was 77 ± 5 years, and sex was equally distributed at baseline (25 females, 25 males). For patient characteristics and coexisting diseases at baseline, see supplementary table 1.

### Clinical outcomes

For the longitudinal outcomes of the clinical parameters, see Table 1. The average Hellström iNPH scale score was 51 ± 12 points preoperatively and 67 ± 15 points at 3 months for the whole cohort. All individual domains of the Hellstrom iNPH scale (gait, balance, neuropsychology and continence) significantly improved at the group level from baseline to 3 months (*p* = 0.001). The gait domain score significantly improved from 40 ± 20 points preoperatively to 63 ± 24 points at 3 months, and this improvement was sustained at 12 and 36 months. The baseline gait velocity for the whole group was 0.64 ± 0.22 m/sec, which significantly improved from 0.93 ± 0.25 at 3 months to 0.94 ± 0.24 at 12 months and was sustained at 0.88 ± 0.27 at 36 months. For individual gait velocity and changes in gait velocity, see Supplementary Figure 1. The MMSE score significantly improved between baseline and 3 months but returned to the preoperative level at 12 months.

**Table 1:** Longitudinal outcome of clinical parameters of whole cohort

	Baseline	3 months	12 months	36 months
Elapsed time from baseline - days		104 ± 17	396 ± 38	1167 ± 115
Assessed patients - no	49	42	43	35
Females – no (%)	25 (51)	20 (48)	21 (49)	21 (60)
Gait velocity – m/sec	0.64 (0.22)	0.93 (0.25) ‡	0.94 (0.24) ‡	0.88 (0.27) ‡
Hellstrom iNPH-scale score				
Total	51 (12)	67 (15) ‡	n/a	n/a
Gait domain	40 (20)	63 (24) ‡	65 (23) ‡	61 (27) ‡
Balance domain	66 (13)	75 (11) ‡	76 (11) ‡	75 (15) ‡
Neuropsychology domain	51 (17)	59 (18) ‡	n/a	n/a
Continence domain	59 (22)	77 (23) ‡	n/a	n/a
10-m time. sec	18 (8.3)	11 (3.5) ‡	11 (3.2) ‡	13 (4.3) ‡
10-m steps	30 (13)	21 (5.3) ‡	20 (4.9) ‡	23 (7.8) ‡
TUG time. sec	25 (16)	14 (5.0) ‡	15 (10) ‡	18 (16) ‡
TUG steps	33 (17)	21 (7.2) ‡	22 (11) ‡	25 (13) ‡
MMSE	25 (2.7)	27 (2.4) ‡	25 (3.2) **	25 (3.4)

Plus–minus values are mean±SD. ‡ significant difference *p* < 0.01 from baseline. † significant difference *p* < 0.05 from baseline. ** significant difference *p* < 0.01 from previous timepoint. * significant difference *p* < 0.05 from previous timepoint

### Volumetric outcomes

The changes in CSF space volume are presented in Table 2. There was a significant reduction in total ventricular volume and individual ventricles from baseline to 3 months (*p* = 0.001), with a continuous trend from 3 to 12 and 36 months, although the difference did not reach statistical significance. All four individual ventricles showed similar decreases over time.

**Table 2:** Longitudinal outcomes of volumetric parameters

	Baseline	3 months	12 months	36 months
Elapsed time from baseline - days		104 ± 17	396 ± 38	1167 ± 115
Assessed patients - n	49	42	43	35
Females – no (%)	25 (51)	20 (48)	21 (49)	21 (60)
Ventricular volume whole group – ml	134 (34)	108 (33) ‡	103 (32) ‡	97 (31) ‡
Ventricular volume females – ml	120 (30)	94 (21) ‡	89(22) ‡	88 (24) ‡
Ventricular volume males – ml	148 (32)	120 (38) ‡	116 (35) ‡	110 (36) ‡
Reduction from baseline whole group - %		20(13)	22(12)	25(14)
Reduction from baseline females - %	N/A	18(9)	22(10) †	25(14) ‡
Reduction from baseline males - %	N/A	21(16)	22(14)	24(13)
Ratio ventricular volume/intracranial volume - whole group	0.089 (0.018)	0.071 (0.018) ‡	0.068 (0.018) ‡	0.065 (0.018) ‡
Ratio ventricular volume/intracranial volume - females	0.084 (0.017)	0.066 (0.013) ‡	0.063 (0.013) ‡	0.062 (0.016) ‡
Ratio ventricular volume/intracranial volume - males	0.094 (0.017)	0.075 (0.021) ‡	0.073 (0.020) ‡	0.070 (0.020) ‡
Lateral ventricles – ml	127 (33)	101 (33) ‡	97 (31) ‡	91 (30) ‡
Third ventricle – ml	4.0 (0.94)	3.5 (0.95) ‡	3.3 (0.80) ‡	3.2 (0.82) ‡
Fourth ventricle – ml	3.2 (1.4)	2.9 (1.2) ‡	2.7 (1.1) ‡	2.6 (1.1) ‡
Intracranial CSF - ml	399 (65)	381 (71) ‡	381.99 (70) ‡	391 (66)
Extraventricular CSF - ml	264 (49)	267 (65)	279 (53) †	294 (49) ‡
Ratio ventricular volume/intracranial CSF	0.34 (0.062)	0.28 (0.060) ‡	0.27 (0.059) ‡	0.25 (0.054) ‡ *
Ratio ventricular volume/extraventricular CSF	0.52 (0.15)	0.40 (0.1205) ‡	0.37 (0.11) ‡	0.33 (0.10) ‡ *
Ratio intracranial CSF/intracranial volume	0.27 (0.031)	0.24 (0.036) ‡	0.25 (0.035) ‡	0.25 (0.034)

The plus–minus values are the mean±SD. ‡ significant difference *p* < 0.01 from baseline. † significant difference *p* < 0.05 from baseline. ** indicates a significant difference (*p* < 0.01) from the previous timepoint. * indicates a significant difference (*p* < 0.05) from the previous timepoint

Twenty-four of the 30 patients who completed all three postoperative assessments did not have any shunt valve adjustment during the study period. In this group, with setting 4 (=110 mm H2O) for 3 years, the ventricular volume significantly decreased from baseline to 3 months (124±29 to 104 ± 23, *p* = 0.001) and from 3 to 12 months (104±23 to 98 ± 26, *p* = 0.025), after which the ventricles were stable (Supplementary Figure 2).

**Fig. 3:**
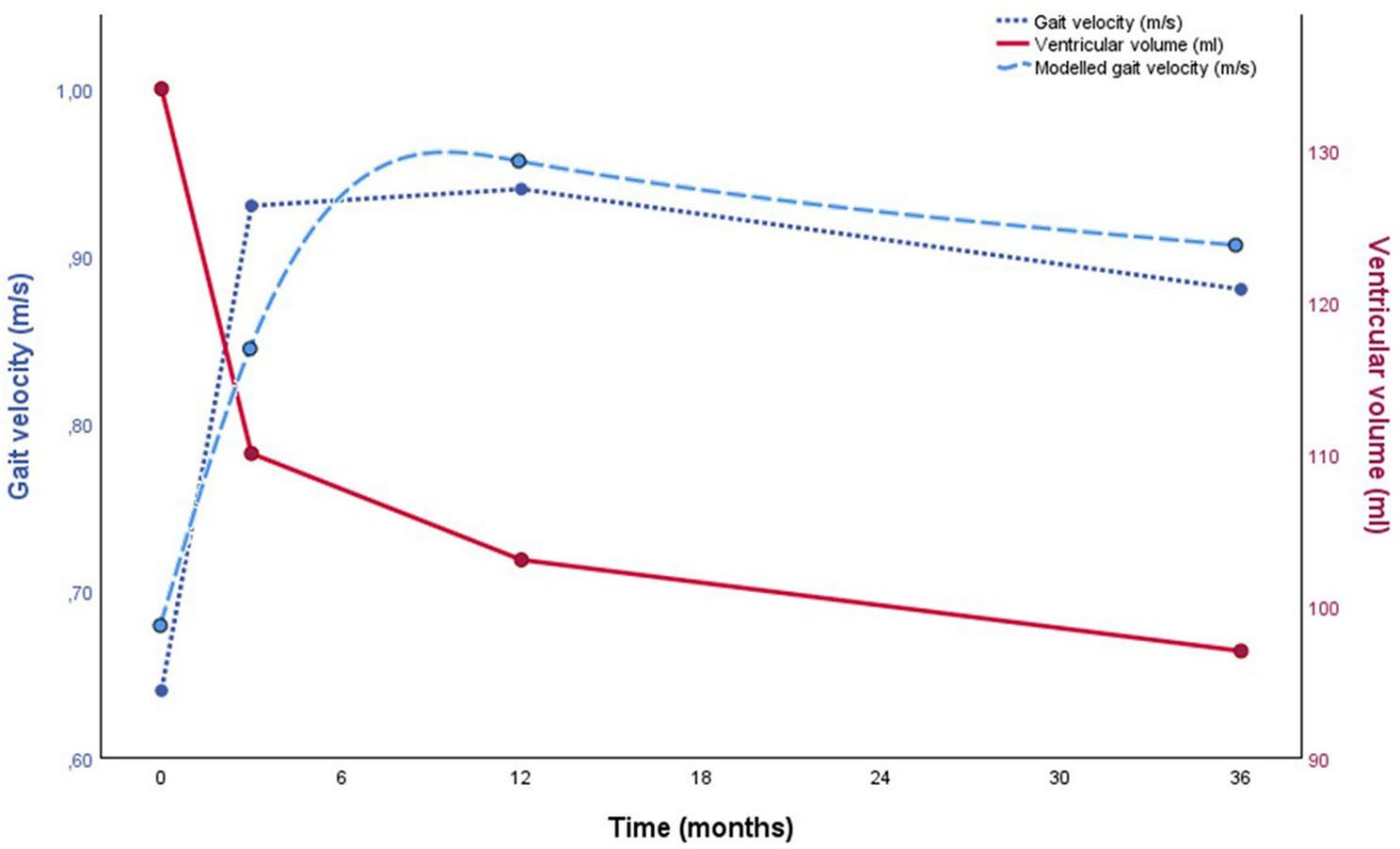
Relationships between gait velocity and volumetric reduction in ventricles from baseline to 36 months. The left (blue) scale and dotted line represent the gait velocity in m/sec. Right (red) scale and full line represent the ventricular volume in ml. The blue interrupted curvilinear line shows the linear mixed model (LMM) prediction of gait velocity (m/s), which explains approximately 35% of the between-measurement variance in gait (pseudo-R^2^), with an intraclass correlation (ICC) of 0.72. The curve shows a modelled peak of gait velocity at 9 months post-operatively

In the LMM, there was a clear complex inverse relationship between gait improvement and ventricular volume. Up to 3 months, reductions in ventricular volume were accompanied by improvements in gait velocity, whereas gait speed subsequently plateaued. The curvilinear form shows that the modelled predicted gait velocity peaks at approximately 9 months and then gradually decreases for the whole cohort (Fig. 3). Larger ventricular volumes were independently associated with slower gait. The inclusion of the quadratic time term (time [2]) captured the observed curvilinear pattern—rapid early gains in gait followed by a plateau and slight attenuation—supporting the use of a polynomial time effect rather than an assumed linear change. The model explained approximately 35% of the between-measures variance in gait (pseudo-R^2^), with a high intraclass correlation (ICC = 0.72). Holding the other terms constant, each 1 ml decrease in ventricular volume was associated with a 0.005 m/s faster gait. Thus, an average reduction of 26–27 ml (≈20%) is associated with an ~0.13 m/s faster gait.

The intracranial CSF volume showed an early reduction that was unchanged at the 12-month follow-up, but at 36 months, it had returned to baseline values. The extraventricular CSF volume increased progressively during the study period, whereas the volume of the ventricles decreased. The ratios of ventricular volume normalized against those of the ICV and intracranial CSF showed a progressive longitudinal reduction for the whole group (Table 2).

### Sex differences in volumetric outcomes

All intracranial and CSF space volumes differed by sex. The intracranial volume at baseline was 1491 ± 131 for the whole group, 1569 ± 123 for males and 1416 ± 91 for females. When normalized to intracranial volume, neither ventricular volume nor intracranial CSF volume differed between the sexes at any time point, except for the ratio of ventricular volume/extraventricular CSF at 12 months. Relative postoperative ventricular volume reduction also did not differ between men and women. Females had significantly worse clinical symptoms at baseline, but there were no sex differences in postoperative clinical parameters (Supplementary Table 2).

### Responders vs nonresponders

At 3 months, 36 out of 42 patients had responded to treatment, equaling a response rate of 86% of the assessed patients. At 12 months, the response rate decreased to 79%, and at 36 months, it decreased to 65%. Clinical responders and nonresponders had similar preoperative clinical symptoms and ventricular volumes (Table 3). However, the volumetric reduction in ventricles was greater (*p* = 0.05) in responders (−28±15 ml or 21%) than in nonresponders (−12±12 ml or 10%) at 3 months. All the nonresponders had their shunt valve setting decreased. At later time points, volumetric reductions were equal between the groups. Pre- and postoperative intracranial CSF, extraventricular CSF and normalized ratios of volumes were also similar at the group level (Supplementary Table 3).

**Table 3:** Differences between responders/nonresponders at 3, 12 and 36 months

Timepoint	3 months		12 months		36 months	
Patients assessed - n	42		43		35	
Outcome	Responders	Nonresponders	Responders	Nonresponders	Responders	Nonresponders
Patients n (%)	36 (86)	6 (14)	34 (79)	9 (21)	23 (65)	12 (35)
Age	77 (5.2)	78 (5.0)	78 (4.7)	80 (4.6)	79 (5.2) †	83 (3.8)
Full iNPH-scale at baseline - points	51 (13)	54 (12)	51 (13)	52 (6.5)	50 (15)	52 (10)
Full iNPH-scale at timepoint - points	69 (14)	57 (12)				
Change in iNPH-scale from baseline - points	18 (8.3) ‡	2.33 (1.37)				
iNPH-scale Gait domain	65 (25)	53 (17)	72 (22) ‡	42 (9.0)	69 (27) ‡	46 (21)
Change in iNPH-scale gait domain from baseline - points	24 (18) ‡	4.0 (4.7)	32 (17) ‡	−0.89 (9.7)	33 (20) ‡	6.2 (17)
Balance domain	76 (12.07)	75 (8.8)	77 (11)	72 (12)	77 (14)	71 (18)
Neuropsychology domain	60(16)	49 (23)				
Continence domain	81 (21.10) ‡	53(24)				
Gait velocity – m/sec	0.95 (0.26)	0.82 (0.16)	1.00 (0.23) ‡	0.71 (0.13)	0.94 (0.28) †	0.75 (0.22)
Gait velocity change compared to baseline – m/sec	0.30 (0.21)‡	0.057 (0.083)	0.38 (0.21) ‡	−0.0015 (0.13)	0.34 (0.23) ‡	0.12 (0.22)
10-m time. sec	11 (3.4)	13 (2.6)	10 (2.7) ‡	15 (3.0)	12 (3.81)	15 (4.7)
10-m steps	21 (5.5)	23 (4.3)	19 (4.0) ‡	26 (3.8)	21 (6.8)	26 (8.6)
TUG time. sec	14 (5.36)	14(2.0)	15 (11.46)	19 (3.8)	18 (19)	19 (7.1)
TUG steps	20 (7.7)	22 (3.9)	20 (12)	28 (5.1)	23 (15)	28 (9.3)
MMSE at baseline - points	25 (2.9)	25 (2.1)	25 (2.8)	28 (5.1)	26 (2.4)	24 (2.5)
MMSE	27 (2.4)	26 (2.1)	26 (3.2) †	23 (2.5)	27 (2.6) †	24 (4.1)
Ventricular volume at baseline - ml	135 (35)	121 (32)	131 (32)	129 (37)	129 (32)	127 (32)
Ventricular volume at timepoint - ml	107 (34)	109 (30)	101 (31)	109 (34)	93 (30)	105 (33)
Reduction of ventricular volume from baseline - ml	28 (15) †	12 (12)	30 (15)	20 (12)	36 (23)	23 (12)
Reduction of ventricular volume from baseline - %	21 (13) †	10 (9.0)	23 (12)	16 (10)	27 (14)	19 (11)

The plus–minus values are the means±SDs. † Significant difference from nonresponders at the 0.05 level. ‡ Significant difference from nonresponders at the 0.01 level

### Shunt adjustments between 3 and 12 months

All patients had a valve setting of 4 (110 mm H₂O resistance) at the first follow-up at 3 months, although two had already undergone additional surgery to add an ASD due to intractable overdrainage symptoms. The remaining patients had no valve adjustments before the first follow-up.

Thirty-nine patients completed both the 3- and 12-month follow-ups and imaging. Ten had their valve settings adjusted by one step between these timepoints, 6 lowered and 4 raised. The ventricular volume in patients whose valve settings were lowered to setting 3 was significantly lower (−12.4±2.6 ml) from 3 to 12 months than that in patients whose valve settings were increased to setting 5 (+1.4±3.2 ml, *p* = 0.007) or unchanged (−4.4±1.2, *p* = 0.03). There was no significant difference between the groups with raised and unchanged valves.

### Overdrainage complications and related symptoms

After 3 months, 12 out of 50 patients had experienced overdrainage-related events or symptoms. Eight patients were treated with valve setting increases alone. Four patients needed additional surgery to add an ASD; among them, two patients who returned within weeks after surgery with intractable overdrainage symptoms and eventually underwent surgery with the addition of an ASD (Shunt Assistant 2.0® 25, Miethke) to allow an open shunt setting at 3 months. At or before the 3-month follow-up, two patients were diagnosed with chronic subdural hematomas, one with bilateral hygromas, a cumulative incidence of subdural effusions of 7%. All subdural effusions were successfully treated by adjusting the Certas valve to setting 8 for a mean of 97 (range 20–166) days. During this time, these patients relapsed in their iNPH-symptoms, but all of them experienced symptom relief after their valves were reopened. However, two patients required an ASD to avoid overdrainage symptoms. At the 3-month follow-up, 8 out of 42 clinically assessed patients reported overdrainage symptoms; all had postural headaches, and most reported one more symptom. These patients had reduced ventricular volumes by 43 ± 18 ml (35% from baseline) compared with patients without complaints (21±12 ml or 16%, *p* < 0.001) (Table 4). Two symptomatic patients had expansive subdural effusions with mass effect. One male with bilateral subdural hematomas had a reduction in baseline ventricular volume of 79 ml or 77%. This patient also accounted for the difference in ventricular reduction between males and females at 3 months. If the two patients with effusions were excluded from the analysis, the remaining six symptomatic patients had a ventricular reduction of 28 ± 11 (*p* = 0.005 compared with asymptomatic patients), while the Evans index did not differ between the groups (0.35±0.03).

**Table 4:** Overdrainage symptoms and volumetric changes at 3 months

	Clinical overdrainage symptoms	No complaints	*p* value
Patients assessed at 3 months–n (%)	8 (19)	34 (81)	<0.001
Symptoms of overdrainage – no of patients	8 Postural headaches	None	
	5 Vertigo		
	5 Tinnitus		
Increase of gait speed – m/s	0.40 (0.31)	0.24 (0.18)	0.059
Reduction of iNPH-scale - points	20 (11)	14 (8.8)	0.16
Evans index at baseline	0.36 (0.018)	0.36 (0.033)	0.83
**Evans index at 3 months**	0.33 (0.0506)	0.37 (0.041)	**0.02**
Ventricular volume at baseline – ml	133(36)	133 (34)	0.94
Ventricular volume at 3 months – ml	89 (45)	112 (29)	0.084
**Reduction of ventricular volume from baseline – ml**	43 (18)	21 (12)	**<0.001**
**Reduction of ventricular volume from baseline – %**	35 (19)	16 (7.9)	**<0.001**
Ratio ventricular volume/intracranial volume at baseline	0.087 (0.018)	0.088 (0.018)	0.83
**Ratio ventricular volume/intracranial volume at 3 months**	0.058 (0.025)	0.074 (0.015)	**0.02**
Ratio ventricular volume/intracranial CSF at baseline	0.36(0.051)	0.33 (0.066)	0.26
Ratio ventricular volume/intracranial CSF at 3 months	0.25 (0.065)	0.28 (0.058)	0.17
Ratio ventricular volume/extraventricular CSF at baseline	0.57 (0.12)	0.51 (0.15)	0.31
Ratio ventricular volume/extraventricular CSF at 3 months	0.347 (0.11)	0.41 (0.12)	0.2

The values are the numbers (%) or means±sds. Patients may have more than one coexisting overdrainage symptom

All patients with clinical overdrainage at three months and no subdural effusion had their valves raised by one setting and became asymptomatic, while they retained subjective iNPH symptom relief at follow up one month later. No patients reported postural headaches at the 12- or 36-month assessments.

### Other complications

Complications up to 3 months included two patients with early postoperative seizures, one with intracerebral hematoma early after surgery, and four with dislocated abdominal catheters. At the 12-month follow-up, one patient was diagnosed with shunt failure, and revision surgery revealed a dysfunctional valve. There were no cases of postoperative infection. After the 12 months follow-up, two patients had bilateral acute subdural hematomas after falls. One patient died, whereas the other patient was successfully treated by adjusting the Certas valve to setting 8/virtual off. Including overdrainage related events, there was a cumulative complication rate in 16 out of 50 patients in the original cohort (32%).

Three patients without symptom improvement were diagnosed with malfunctioning shunts, as ventricular volumes remained unchanged from the preoperative baseline values (Supplementary Figure 3). At 3 months, one male had been discharged after surgery with a shunt valve accidentally set to 8/virtual off by a junior colleague, and one female had a dislocated abdominal catheter, which was discovered after the volumetric analysis. Another female experienced symptom relapse at the 12-month follow-up, with ventricular volume returning to preoperative levels after a previous marked reduction at 3 months. Surgical revision confirmed a dysfunctional valve, the only one identified over more than 110 shunt years in this study. The patients’ symptoms improved along with a decrease in ventricular volume, without overdrainage symptoms. None of these patient timepoints were included in the data analysis, as the shunts were malfunctioning.

## Discussion

This study provides one of the longest structured, prospective follow-up studies of ventricular volumetry in iNPH, offering insights into long-term clinical and radiological outcomes after shunting. The ventricular volume decreased substantially by 3 months, indicating an early steep reduction followed by a prolonged shrinkage phase. Much of the ventricular reduction occurred in the first 36 hours after surgery, according to our earlier analysis of this same cohort [8]. Our findings confirm that all individual ventricles decreased in volume and are in line with the findings of Cogswell et al. [20] and Yamada et al. [13], who reported early postoperative reductions. However, interindividual variation among patients with the same shunt setting was substantial.

The progressive reduction in extraventricular CSF along with the transient reduction in intracranial CSF volume also aligns with observations reported by Yamada [13]. We interpret these results as indicating that shunt surgery effectively decompressed the tight high-convexity sulci through decreased ventricular volume, moving the iNPH-affected brain away from the DESH-phenotype and allowing it to regain a more normal shape. The normalized ratio of ventricular volume to intracranial CSF may be regarded as a “3D-Evans index”, with values similar to the original index but greater sensitivity to postoperative change. While male and female patients showed the expected difference in absolute volume, the normalized ratios did not show any relevant sex-related differences.

Volumetry did not reliably predict outcomes in this study, as preoperative volumes did not distinguish responders from nonresponders. Postoperatively, however, responders showed greater reductions at early follow-up at 3 months, which is consistent with the findings of Neikter et al. [7]. Our results and those of Luciano et al. [11] suggest that a mean reduction of ≈20% of baseline (approx. 27–30 ml in males, 21–24 ml in females in this cohort) is needed for early clinical improvement, although this alone is not sufficient to guarantee a response. When clinical improvement is absent, failure to achieve early ≈20% volumetric reduction could prompt physicians to consider shunt malfunction or to optimize valve settings to exclude underdrainage as a cause. The lack of between-group volumetric differences at the 12- and 36-month time points—after valve optimization—likely reflects a greater proportion of true nonresponders. Over time, with progressive age and added comorbidities, the relationship between CSF space volume and symptoms became less clear, often because of locomotor system comorbidities, progressive neurodegenerative brain disorders with atrophy, or a renewed need to further reduce valve resistance to sustain the treatment effect. Over the course of the study period, the responder rate decreased from 86% at 3 months to 65% at 36 months, when nonresponders were significantly older. Few patients with clinical deterioration contacted our department and were identified only at follow-up as having relative underdrainage or even shunt failure, prompting a reduction in valve setting or shunt revision. These findings also indicate the need for an active volumetric and clinical follow-up program at regular intervals to monitor treatment effects and optimize valve settings, as iNPH patients may not always be aware of clinical deterioration.

Overdrainage can result in serious complications, such as subdural hematomas and neurological deficits [21]. Overdrainage complications and symptoms up to 3 months were frequent (24%) in our cohort and were associated with greater reductions (35%) in ventricular volume. With an applicable method for ventricular volumetry, such as the one used in this study, clinicians could balance efficacy and risk and fine tune valve settings and hence reduce the ventricle size to be significant but not approach 30% of the initial volume. Linear indices (frontal horn width, EI) often change only discretely in overshunted patients without subdural effusions, also in our sample, whereas volumetry appears to offer the precision needed for confirming overdrainage and guiding adjustments [7, 15, 22].

The choice of valve design influences overdrainage: any differential-pressure (DP-)valve without anti-siphoning/gravitational protection increases the risk [23, 24] and the evidence favouring integrated ASDs in shunt systems is comprehensive [9, 24–28]. Among individuals with ASDs, gravitational valves have been shown to have a more favourable effect on overdrainage symptoms than flow reducers have in larger cohort studies [11, 24]. In our cohort, 4 out of 50 patients required secondary ASD insertion. Given the local costs (integrated SiphonGuard® ≈ USD 240; revision surgery ≈ USD 6800), primary ASD implantation in all 50 patients in this cohort would have been cost-effective at a conversion threshold of only 1.73 patients. The cost of all outpatient consultations for valve adjustments and radiological follow-up is not included in this calculation. Until the study was conducted, DP-valves without ASDs were the default in our department’s hydrocephalus practice. Since the study results and high frequency of overdrainage-related events became apparent, we have changed our department’s clinical routine and now implant Certas Plus® with an integrated SiphonGuard at an initial setting of 5 (=140 mm H₂O) in iNPH. In our overall hydrocephalus practice, only patients with extreme low-pressure hydrocephalus and mainly bedridden patients have a reason not to have an ASD integrated into their shunt system.

Valve adjustments were common: 12 patients had valve settings that changed at or after 3 months, and an additional 5 had their valve settings lowered at 12–36 months for symptom relapse. None of these patients reported overdrainage symptoms after the latter adjustments despite having ventricular volumes associated with overdrainage earlier in the postoperative course, suggesting greater sensitivity to overdrainage in the first 3 months after surgery—which is consistent with Yamada’s findings [29]. These results suggest that patients with late symptom relapse safely can and should have their valve setting lowered to assess symptom reversibility, as proposed. All nonresponders without other obvious comorbidities explaining their deterioration were offered a reduction in valve setting by one or more steps and were followed up by telephone interview. While not all patients accepted this adjustment because of fear of overdrainage events, our experience aligns with the observation by Gutowski et al. [30] that only approximately one-fourth of patients with secondary deterioration benefit from reducing the valve setting.

Volumetry also aided in shunt evaluation: three malfunctions were identified as deviations from expected volumetric trajectories, suggesting that volumetry could be used as a noninvasive method to assess shunt function. Establishing a preoperative baseline and/or a postoperative baseline after 3 months—when ventricles have reduced substantially and the patient is clinically improved—provides a reliable reference for future evaluations when new symptoms of possible shunt failure arise. In our cohort, lowering valve resistance resulted in significantly greater reductions in ventricular volume than in increasing it, which showed a surprisingly small expansion of ventricles. When shunt function is being assessed, a significant decrease in volume after lowering valve resistance could support patency, whereas a lack of change should prompt further invasive evaluation.

Clinically, shunting produced robust motor gains; most patients substantially exceeded the 0.10 m/s minimum meaningful change in gait velocity in older adults [31], which aligns with prior literature [11]. These motor effects were sustained at long-term follow-up. According to the LMM, a typical 26–27 ml (≈20%) ventricular reduction at 3 months predicts ~0.13 m/s faster gait. The model explained ~35% of the gait change, while comorbidities such as neurodegeneration and musculoskeletal disease also influenced recovery. Improvements in cognition and continence at the group level were clear at 3 months. Although our study did not include advanced longitudinal monitoring of these symptoms beyond 3 months, the 12-month decrease in the MMSE score suggests a transient cognitive benefit. Although shunting addresses the mechanical issue of impaired CSF drainage—possibly contributing more to motor symptoms—it does not mitigate other pathophysiological processes present in iNPH. The sex difference in preoperative symptom burden in this cohort confirms the findings of previous studies from a European multicenter cohort [32] and the Swedish hydrocephalus registry [33, 34]; women are diagnosed and treated later, at a more advanced stage of the disease. Given that the sex distribution was equal in this prospective cohort, we suspect a sex-related delay in diagnosis among women. The reasons for this deserve to be studied more closely.

The complication rates for subdural effusions were comparable to those in previous reports [21, 35]. An unusually high rate of peritoneal catheter extrusion has led to a change in local practice; our department now uses laparoscopy-assisted or superior right subcostal entry with subfascial distal tunneling before intraabdominal placement and slow-resorbable monofilament fascial closure.

Future studies are needed to assess the generalizability of our findings and should include larger multicenter cohorts to validate automated volumetry across scanners, software, and valve types and centers. Study protocols combining volumetry with MR elastography may clarify interindividual compliance and drainage dynamics [36]. Volumetric assessment before and after valve adjustment in a randomized, blinded study setting could lead to the evaluation of volumetry as a tool for assessing shunt patency. Additionally, a comparison of volumetric changes in relation to novel 2D linear measures such as the z-Evans index [12], callosal angle, tight high convexity sulcus and other domains of the iNPH Radscale [37] in a similar cohort is needed to establish the role of volumetry in the longitudinal follow-up of these patients. Finally, other volumetric changes in the brain or CSF and measures outside the ventricles, such as the qDESH [38], DESH- Venthi and Sylhi indices [13], need to be explored in a longitudinal patient cohort of shunted iNPH patients. These are not available in the SyMRI software and were hence not included in this study.

This study has several other limitations. Despite consecutive and prospective recruitment, the single-center design introduces a risk of selection bias. The use of only one type of MRI scanner and shunt valve limits the generalizability of the findings to specifically Codman Certas valves without ASDs. For several reasons, the MMSE score is not an optimal marker of cognitive decline in patients with iNPH when used alone [39, 40]. The absence of comprehensive neuropsychological testing at 12 and 36 months makes the cognitive outcomes at these later time points uncertain. We chose to use the full iNPH-scale when available at 3 months and the iNPH-scale gait domain as the responder measure at the remaining time points. This makes the focus on motor symptoms stronger at later time points and could affect longitudinal comparisons of responder rates over time. Additionally, the small size of this baseline cohort and the progressively smaller number of observations over time make the findings less reliable in later phases of follow-up. For example, several patients with severe cognitive decline during the study period could not complete the 36-month follow-up for MRI and clinical assessment. While the study was adequately powered for the primary within-patient volumetric endpoint, secondary and subgroup analyses were relatively small and should be interpreted as hypothesis-generating. Finally, we did not have the resources to perform invasive infusion testing to determine shunt function in all the patients at all the study follow-up time points [41].

**Fig. 4:**
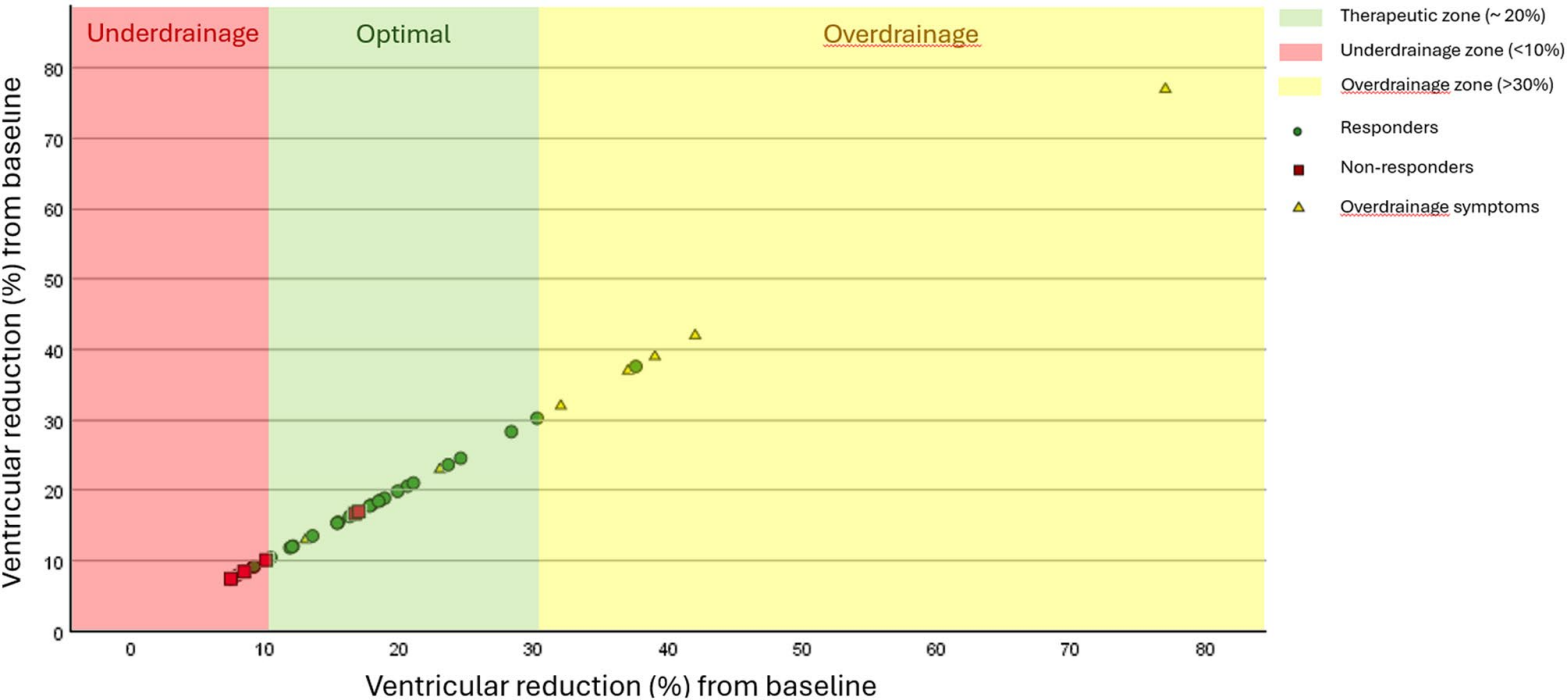
A hypothetical window for early ventricular reduction 3 months after shunting in iNPH patients. Diagram illustrating proposed approximate zones of under- and overdrainage risk and a theoretical therapeutic window for optimal ventricular volume reduction associated with clinical response without symptomatic overdrainage. Seven of the eight patients with overdrainage symptoms were also responders

Despite these limitations, the clinical and radiological findings from this prospective cohort of iNPH patients contribute to a growing body of studies aiming to establish evidence-based decision thresholds by linking relative ventricular volumetric reductions with clinical response and overdrainage. The relative reduction of ventricles in percent from baseline is an intuitive and easy parameter for clinicians and radiologists to calculate and relate to. Additionally, as the patient provides its own baseline reference, there is no need to normalize against intracranial volume. The development of clinical protocols for routine postoperative volumetric assessments at standardized intervals could further improve shunt therapy in iNPH patients. In this cohort, at least at the early 3-month follow-up after shunt implantation, we observed that an approximately 20% decrease in ventricular volume appears to be a practical target that maximizes benefit while limiting overdrainage. We hypothesize that there may be a “volumetric therapeutic window” between under- and overdrainage that should be explored further (Fig. 4). Within a personalized medicine framework, volumetric surveillance could guide valve adjustments to keep patients above a 10% reduction, near a 20% reduction, and below a 30% reduction from baseline. This concept should at this point be regarded as strictly exploratory and requires validation in larger multicenter cohorts with standardized valve adjustment protocols, including different shunt valves and ASDs. 

In conclusion, we report that ventricular volumetry is an efficient, patient- and user-friendly method for longitudinal monitoring of shunt response in iNPH patients. With the valve setting unchanged, most of the ventricular volume reduction occurs within the first three months after surgery but continues at a slower rate up to 12 months. We found a difference in the rate of ventricular reduction between responders and nonresponders at 3 months but not thereafter. A 20% reduction in baseline ventricular volume appears to be an optimal target for achieving a clinical response at 3 months after surgery. Overdrainage after shunting in iNPH patients is common, and an ASD should be considered for all patients from the beginning. Overdrained patients with postural headaches presented a mean reduction in ventricular volume of more than 30% of baseline. Reductions following a decrease in shunt valve resistance are readily detectable by ventricular volumetry, supporting its use as a noninvasive method to evaluate shunt function.

## Electronic supplementary material

Below is the link to the electronic supplementary material.

Supplementary material 1

## Data Availability

The data that support the findings of this study are not openly available due to reasons of sensitivity and are available from the corresponding author upon reasonable request. Data are located in controlled access data storage at the Department of neurology, University Hospital Linköping, Sweden.

## Declarations

### Presentation

Preliminary results from this study were presented at Hydrocephalus 2024, the annual meeting of the International Society of Hydrocephalus and Cerebrospinal Fluid Disorders.

## Abbreviations

iNPH: Idiopathic Normal Pressure Hydrocephalus
MMSE: Mini-Mental State Examination
EI: Evans Index
MRI: Magnetic Resonance Imaging
qMRI: Quantitative MRI
2D: Two-dimensional
3D: Three-dimensional
3T: 3 Tesla
3D-QALAS: 3D “quantification using an interleaved Look–Locker acquisition sequence”
TFE: Turbo Field Echo
ICV: Intracranial volume
ASD: Antisiphoning device
ANOVA: Analysis of Variance
rmLMM: Repeated-measures Linear Mixed Model
CSF: Cerebrospinal Fluid
ICP: Intracranial Pressure
USD: United States Dollar
AB: Aktiebolag (Swedish “limited company”)
ml: Milliliter(s)
mm Hg: Millimeters of mercury
mm H_2_O: Millimeters of water
m/s: Meters per second
m: Meter(s)
sec: Second(s)
SD: Standard deviation

## References

[1] Adams RD, Fisher CM, Hakim S, Ojemann RG, Sweet WH. Symptomatic occult hydrocephalus with “normal” cerebrospinal-fluid pressure. N Engl J Med. 1965 Jul 15;273(3):117–26. 10.1056/nejm196507152730301. 14303656 10.1056/NEJM196507152730301

[2] Sundstrom N, Malm J, Laurell K, Lundin F, Kahlon B, Cesarini KG, et al. Incidence and outcome of surgery for adult hydrocephalus patients in Sweden. Br J Neurosurg. 2017 Feb;31(1):21–27. 10.1080/02688697.2016.1229749. 27619731 10.1080/02688697.2016.1229749

[3] Constantinescu C, Wikkelsø C, Westman E, Ziegelitz D, Jaraj D, Rydén L, et al. Prevalence of possible idiopathic normal pressure hydrocephalus in Sweden: a population-based MRI study in 791 70-year-old participants. Neurology. 2024 Jan 23;102(2):e208037. 10.1212/wnl.0000000000208037. 10.1212/WNL.0000000000208037 PMC10962905 38165321

[4] Toma AK, Holl E, Kitchen ND, Watkins LD. Evans’ index revisited: the need for an alternative in normal pressure hydrocephalus. Neurosurgery. 2011 Apr;68(4):939–44. 10.1227/NEU.0b013e318208f5e0. 21221031 10.1227/NEU.0b013e318208f5e0

[5] Ambarki K, Israelsson H, Wahlin A, Birgander R, Eklund A, Malm J. Brain ventricular size in healthy elderly: comparison between Evans index and volume measurement. Neurosurgery. 2010 Jul;67(1):94–99; discussion 99. 10.1227/01.neu.0000370939.30003.d1. 20559096 10.1227/01.NEU.0000370939.30003.D1

[6] Holmgren RT, Tisell A, Warntjes MJB, Georgiopoulos C. 3D quantitative MRI: a fast and reliable method for ventricular volumetry. World Neurosurg. 2025 Feb 3;195:123661. 10.1016/j.wneu.2025.123661. 10.1016/j.wneu.2025.123661 39788420

[7] Neikter J, Agerskov S, Hellström P, Tullberg M, Starck G, Ziegelitz D, et al. Ventricular volume is more strongly associated with clinical improvement than the Evans index after shunting in idiopathic normal pressure hydrocephalus. AJNR Am J Neuroradiol. 2020 Jul;41(7):1187–92. 10.3174/ajnr.A6620. 32527841 10.3174/ajnr.A6620 PMC7357646

[8] Holmgren RT, Nilsson M, Georgiopoulos C, Zsigmond P. A randomized Double-blinded clinical study of early volumetric changes after shunt surgery and MRI-Resistance of the Codman Certas® Plus shunt valve. World Neurosurg. 2025 Aug 29;202:124424. 10.1016/j.wneu.2025.124424. 40887008 10.1016/j.wneu.2025.124424

[9] Kelbert J, Nosova K, Kern A, Russell R, Pico A, Mamaril-Davis J, et al. Idiopathic normal pressure hydrocephalus and shunt complications per valve type: a meta-analysis of proportions. World Neurosurg. 2025 Feb;194:123450. 10.1016/j.wneu.2024.11.033. 39577651 10.1016/j.wneu.2024.11.033

[10] Ros B, Iglesias S, Linares J, Cerro L, Casado J, Arráez MA. Shunt overdrainage: reappraisal of the syndrome and proposal for an integrative model. JCM. 2021 Aug 17;10(16):3620. 10.3390/jcm10163620. 34441916 10.3390/jcm10163620 PMC8396927

[11] Luciano MG, Williams MA, Hamilton MG, Katzen HL, Dasher NA, Moghekar A, et al. A randomized trial of shunting for idiopathic normal-pressure hydrocephalus. N Engl J Med. 2025 Sep 16;393(22):2198–209. 10.1056/NEJMoa2503109. 40960253 10.1056/NEJMoa2503109 PMC12682072

[12] Yamada S, Ishikawa M, Yamamoto K. Optimal diagnostic indices for idiopathic normal pressure hydrocephalus based on the 3D quantitative volumetric analysis for the cerebral ventricle and subarachnoid space. AJNR Am J Neuroradiol. 2015 Dec;36(12):2262–69. 10.3174/ajnr.A4440. 26359148 10.3174/ajnr.A4440 PMC7964275

[13] Yamada S, Ishikawa M, Yamaguchi M, Yamamoto K. Longitudinal morphological changes during recovery from brain deformation due to idiopathic normal pressure hydrocephalus after ventriculoperitoneal shunt surgery. Sci Rep. 2019 Nov 21;9(1):17318. 10.1038/s41598-019-53888-7. 31754171 10.1038/s41598-019-53888-7 PMC6872815

[14] Marmarou A, Bergsneider M, Relkin N, Klinge P, Black PM. Development of guidelines for idiopathic normal-pressure hydrocephalus: introduction. Neurosurgery. 2005 Sep;57(suppl_3):SS2–3; discussion ii-v. 10.1227/01.neu.0000168188.25559.0e. 10.1227/01.neu.0000168188.25559.0e 16160424

[15] Lidén S, Farahmand D, Laurell K. Volumetric effect of shunt adjustments in normal pressure hydrocephalus: a randomized, double-blind trial. J Neurosurg. 2023 Nov 17;1–8. 10.3171/2023.9.Jns23668. 10.3171/2023.9.JNS23668 37976516

[16] Ziegelitz D, Hellström P, Björkman-Burtscher IM, Agerskov S, Stevens-Jones O, Farahmand D, et al. Evaluation of a fully automated method for ventricular volume segmentation before and after shunt surgery in idiopathic normal pressure hydrocephalus. World Neurosurg. 2024 Jan;181:e303–11. 10.1016/j.wneu.2023.10.045. 10.1016/j.wneu.2023.10.045 37838163

[17] Hellstrom P, Klinge P, Tans J, Wikkelso C. A new scale for assessment of severity and outcome in iNPH. Acta Neurol Scand. 2012 Oct;126(4):229–37. 10.1111/j.1600-0404.2012.01677.x. 22587624 10.1111/j.1600-0404.2012.01677.x

[18] Folstein MF, Folstein SE, McHugh PR. “Mini-mental state”. A practical method for grading the cognitive state of patients for the clinician. J Psychiatric Res. 1975 Nov;12(3):189–98. 10.1016/0022-3956(75)90026-6. 10.1016/0022-3956(75)90026-6 1202204

[19] Fujita S, Hagiwara A, Hori M, Warntjes M, Kamagata K, Fukunaga I, et al. Three-dimensional high-resolution simultaneous quantitative mapping of the whole brain with 3D-QALAS: an accuracy and repeatability study. Magn Reson Imag. 2019 Nov;63:235–43. 10.1016/j.mri.2019.08.031. 10.1016/j.mri.2019.08.031 31445118

[20] Cogswell PM, Murphy MC, Senjem ML, Botha H, Gunter JL, Elder BD, et al. Changes in ventricular and cortical volumes following shunt placement in patients with idiopathic normal pressure hydrocephalus. AJNR Am J Neuroradiol. 2021 Dec;42(12):2165–71. 10.3174/ajnr.A7323. 34674997 10.3174/ajnr.A7323 PMC8805754

[21] Giordan E, Palandri G, Lanzino G, Murad MH, Elder BD. Outcomes and complications of different surgical treatments for idiopathic normal pressure hydrocephalus: a systematic review and meta-analysis. J Neurosurg. 2019 Oct 1;131(4):1024–36. 10.3171/2018.5.Jns1875. 30497150 10.3171/2018.5.JNS1875

[22] Virhammar J, Laurell K, Cesarini KG, Larsson EM. Increase in callosal angle and decrease in ventricular volume after shunt surgery in patients with idiopathic normal pressure hydrocephalus. J Neurosurg. 2018 Jan 1;130(1):130–35. 10.3171/2017.8.jns17547. 29393749 10.3171/2017.8.JNS17547

[23] Czosnyka Z, Czosnyka M, Richards HK, Pickard JD. Posture-related overdrainage: comparison of the performance of 10 hydrocephalus shunts in vitro. Neurosurgery. 1998 Feb;42(2):327–34; discussion 333-4. 10.1097/00006123-199802000-00069. 9482183 10.1097/00006123-199802000-00069

[24] Lemcke J, Meier U, Müller C, Fritsch MJ, Kehler U, Langer N, et al. Safety and efficacy of gravitational shunt valves in patients with idiopathic normal pressure hydrocephalus: a pragmatic, randomised, open label, multicentre trial (SVASONA). J Neurol, Neurosurg Psychiatry. 2013 Aug;84(8):850–57. 10.1136/jnnp-2012-303936. 23457222 10.1136/jnnp-2012-303936 PMC3717598

[25] Panagopoulos D, Stranjalis G, Gavra M, Boviatsis E, Korfias KSS. Shunt over-drainage, Slit ventricle Syndrome, Programmable valves and Anti-Siphon Devices. A Narrative review of a Multifactorial and intractable Problem. J Integr Neurosci. 2022 Apr 18;21(3):84. 10.31083/j.jin2103084. 35633165 10.31083/j.jin2103084

[26] Huang AP, Kuo LT, Lai DM, Yang SH, Kuo MF. Antisiphon device: a review of existing mechanisms and clinical applications to prevent overdrainage in shunted hydrocephalic patients. Biomed J. 2022 Feb;45(1):95–108. 10.1016/j.bj.2021.08.001. 34411787 10.1016/j.bj.2021.08.001 PMC9133390

[27] Gutowski P, Gölz L, Rot S, Lemcke J, Thomale UW. Gravitational shunt valves in hydrocephalus to challenge the sequelae of over-drainage. Expert Rev Med Devices. 2020 Nov;17(11):1155–68. 10.1080/17434440.2020.1837622. 33176494 10.1080/17434440.2020.1837622

[28] Meier U, Stengel D, Müller C, Fritsch MJ, Kehler U, Langer N, et al. Predictors of subsequent overdrainage and clinical outcomes after ventriculoperitoneal shunting for idiopathic normal pressure hydrocephalus. Neurosurgery. 2013 Dec;73(6):1054–60. 10.1227/neu.0000000000000155. 24257332 10.1227/NEU.0000000000000155

[29] Yamada S, Ishikawa M, Nakajima M, Nozaki K. Reconsidering ventriculoperitoneal shunt surgery and postoperative shunt valve pressure adjustment: our approaches learned from past challenges and failures. Front Neurol. 2022;12:798488. 10.3389/fneur.2021.798488. 35069426 10.3389/fneur.2021.798488 PMC8770742

[30] Gutowski P, Rot S, Fritsch M, Meier U, Gölz L, Lemcke J. Secondary deterioration in patients with normal pressure hydrocephalus after ventriculoperitoneal shunt placement: a proposed algorithm of treatment. Fluids Barriers CNS. 2020 Mar 4;17(1):18. 10.1186/s12987-020-00180-w. 32127017 10.1186/s12987-020-00180-w PMC7055114

[31] Perera S, Mody SH, Woodman RC, Studenski SA. Meaningful change and responsiveness in common physical performance measures in older adults. J Am Geriatrics Soc. 2006 May;54(5):743–49. 10.1111/j.1532-5415.2006.00701.x. 10.1111/j.1532-5415.2006.00701.x 16696738

[32] Klinge P, Hellstrom P, Tans J, Wikkelso C. One-year outcome in the European multicentre study on iNPH. Acta Neurol Scand. 2012 Sep;126(3):145–53. 10.1111/j.1600-0404.2012.01676.x. 22571428 10.1111/j.1600-0404.2012.01676.x

[33] Sundström N, Lundin F, Arvidsson L, Tullberg M, Wikkelsø C. The demography of idiopathic normal pressure hydrocephalus: data on 3000 consecutive, surgically treated patients and a systematic review of the literature. J Neurosurg. 2022 Nov 1;137(5):1310–20. 10.3171/2022.2.Jns212063. 35395629 10.3171/2022.2.JNS212063

[34] Chidiac C, Sundström N, Tullberg M, Arvidsson L, Olivecrona M. Waiting time for surgery influences the outcome in idiopathic normal pressure hydrocephalus — a population-based study. Acta neurochirurgica. 2022 Feb;164(2):469–78. 10.1007/s00701-021-05085-7. 34970701 10.1007/s00701-021-05085-7 PMC8854261

[35] Sundstrom N, Lagebrant M, Eklund A, Koskinen L-OD, Malm J. Subdural hematomas in 1846 patients with shunted idiopathic normal pressure hydrocephalus: treatment and long-term survival. J Neurosurg. 2018 Sep;129(3):797–804. 10.3171/2017.5.jns17481. 29076787 10.3171/2017.5.JNS17481

[36] Karki P, Murphy MC, Cogswell PM, Senjem ML, Graff-Radford J, Elder BD, et al. Prediction of surgical outcomes in normal pressure hydrocephalus by MR Elastography. AJNR Am J Neuroradiol. 2024 Mar 7;45(3):328–34. 10.3174/ajnr.A8108. 38272572 10.3174/ajnr.A8108 PMC11286123

[37] Kockum K, Lilja-Lund O, Larsson EM, Rosell M, Söderström L, Virhammar J, et al. The idiopathic normal-pressure hydrocephalus Radscale: a radiological scale for structured evaluation. Euro J Neurol. 2018;25(3):569–76. 10.1111/ene.13555. 10.1111/ene.13555 29281156

[38] Behndig S, Lalou A, Axelsson J, Larsson J, Wåhlin A, Ryska P, et al. qDESH: a method to quantify disproportionately enlarged subarachnoid space hydrocephalus. Fluids Barriers CNS. 2025 Jul 1;22(1):67. 10.1186/s12987-025-00677-2. 40597328 10.1186/s12987-025-00677-2 PMC12219777

[39] Hensel A, Angermeyer MC, Riedel-Heller SG. Measuring cognitive change in older adults: reliable change indices for the Mini-Mental state Examination. J Neurol, Neurosurg Psychiatry. 2007 Dec;78(12):1298–303. 10.1136/jnnp.2006.109074. 17442763 10.1136/jnnp.2006.109074 PMC2095596

[40] Langheinrich T, Chen C, Thomas O. Update on the cognitive presentations of iNPH for clinicians. Front Neurol. 2022;13:894617. 10.3389/fneur.2022.894617. 35937049 10.3389/fneur.2022.894617 PMC9350547

[41] Eklund A, Lundkvist B, Koskinen LO, Malm J. Infusion technique can be used to distinguish between dysfunction of a hydrocephalus shunt system and a progressive dementia. Med Biol Eng Comput. 2004 Sep;42(5):644–49. 10.1007/bf02347546. 15503965 10.1007/BF02347546

