# Supplementary material for: Ventricular volumetry in relation to clinical response and overdrainage after shunt surgery in idiopathic normal-pressure hydrocephalus: a three-year prospective study
Source: Fluids Barriers CNS. 2026 Jun 30;23:86. doi: 10.1186/s12987-026-00835-0 (PMC13321627; doi:10.1186/s12987-026-00835-0)
Supplement: Supplementary file 1 — Supplementary material 1 [file 12987_2026_835_MOESM1_ESM.docx]

**Supplementary Table 1 – Patient characteristics and coexisting diseases at baseline for the whole cohort of 50 patients**

Values are no (%) or means±SDs. Patients may have more than one coexisting disease.

| **Demographic and radiological characteristics at baseline - mean±SD** | |
| --- | --- |
| Male/female | 25/25 |
| Age at surgery - yrs | 77.1±5.0 |
| Duration of iNPH-symptoms - months | 33±27 |
| Intracranial volume - ml | 1491±132 |
| Evan´s index at baseline | 0.36±0.035 |
| Callosal angle at baseline | 68±15 |
| Radscale score at baseline | 8.1±1.7 |
| Total intracranial CSF - ml | 399±65 |
| **Coexisting diseases at baseline – no (%)** | |
| Free from any coexisting disease | 6 (12) |
| History of myocardial infarction or angina | 13 (26) |
| Atrial fibrillation | 4 (8) |
| Hypertonia | 35 (71) |
| History of cerebrovascular disease | 17 (35) |
| History of other neurological illness | 8 (16) |
| Dementia | 3 (6) |
| Diabetes | 9 (18) |
| Polyneuropathy | 2 (4) |
| History of psychosis | 1 (2) |
| History of depression | 10 (20) |
| Hyperlipaemia | 29 (59) |
| Use of oral anticoagulants | 25 (51) |
| Clopidogrel | 4 (8) |
| Direct oral anticoagulant (DOAC) | 2 (4) |
| Acetylsalicylic acid | 15 (31) |
| Warfarin | 3 (6) |
| Musculoskeletal conditions impacting gait | 18 (37) |
| Lumbar spinal stenosis | 7 (14) |
| Arthritis of hip or knee | 9 (18) |
| Active smokers | 9 (18) |
| Heredity for hydrocephalus | 4 (8) |

**Supplementary Table 2. Sex differences in clinical and volumetric parameters**

The values are the mean±SD. ‡ significant difference p<0.01 compared with females. † significant difference p<0.05 compared with females.

| **Timepoint** | **Baseline** | | **3 months** | | **12 months** | | **36 months** | |
| --- | --- | --- | --- | --- | --- | --- | --- | --- |
| Patients | 49 | | 42 | | 43 | | 35 | |
| Male/female | 24 | 25 | 22 | 20 | 22 | 21 | 14 | 21 |
| Age | 77 (4.1) | 77 (6.0) | 77 (4.1) | 76 (6.1) | 78 (4.1) | 79 (5.4) | 80 (4.6) | 80 (5.5) |
|  |  |  |  |  |  |  |  |  |
| **Clinical parameters** |  |  |  |  |  |  |  |  |
| Full iNPH-scale at baseline - points | 55 (12) † | 47 (11) | 55 (13) | 48 (12) | 54 (12) | 48 (12) | 57 (13) | 47 (12) |
| Full iNPH-scale at timepoint - points | 55 (12) † | 47 (11) | 70 (15) | 64 (14) | n/a | n/a | n/a | n/a |
| Change in iNPH-scale from baseline - points |  |  | 15 (10) | 16 (8.9) | n/a | n/a | n/a | n/a |
| iNPH-scale Gait domain | 48 (19) ‡ | 32 (17) | 69 (23) | 56 (23) | 71 (22) | 60 (23) | 66 (30) | 57 (25) |
| Change in iNPH-scale gait domain from baseline - points |  |  | 20 (18) | 22 (18) | 24 (24) | 27 (18) | 19 (25) | 26 (21) |
| Balance domain | 70 (10.48) | 64 (13.52) | 77 (13) | 73 (9.7) | 79 (13) | 73 (9.6) | 78 (18) | 72 (13) |
| Neuropsychology domain | 48 (20) | 55 (14) | 57 (18) | 61(17) | n/a | n/a | n/a | n/a |
| Continence domain | 62 (21) | 56 (24) | 79 (20) | 74 (27) | n/a | n/a | n/a | n/a |
|  |  |  |  |  |  |  |  |  |
| Gait velocity – m/sec | 0.71 (0.21) † | 0.58 (0.21) | 0.99 (0.27) | 0.86 (0.2193) | 1.0 (0.25) | 0.87 (0.22) | 0.93 (0.31) | 0.83 (0.24) |
| Gait velocity change compared to baseline – m/sec |  |  | 0.28 (0.24) | 0.25 (0.20) | 0.31 (0.29) | 0.29 (0.21) | 0.25 (0.24) | 0.28 (0.25) |
| 10-m time. sec | 16 (5.6) † | 20 (9.9) | 11 (3.6) | 12 (3.2) | 10 (2.6) | 12 (3.6) | 12 (4.2) | 13 (4.3) |
| 10-m steps | 26 (8.8) † | 34 (15) | 20 (5.3) | 23 (5) | 19 (4.5) | 22 (50) | 21 (7.7) | 24 (7.8) |
| TUG time. sec | 20 (10) † | 30 (18) | 13 (4.9) | 15 (5.1) | 13 (3.9) | 18 (14) | 15 (5.6) | 21 (20) |
| TUG steps | 29 (17) | 37 (17) | 19 (6.5) | 23 (7.7) | 19 (6.3) | 25 (14) | 22 (8.7) | 27 (15) |
| MMSE at baseline - points | 25 (2.9) | 26 (2.5) | 25 (3) | 26 (2.3) | 24 (3) | 26 (2.3) | 25 (2.8) | 26 (2.4) |
| MMSE | 25 (2.9) | 26 (2.5) | 27 (2.8) | 27 (1.8) | 25 (3.4) | 25 (3) | 26 (3.6) | 25 (3.3) |
|  |  |  |  |  |  |  |  |  |
| **Volumetric parameters** |  |  |  |  |  |  |  |  |
| Ventricular volume at baseline - ml | 148 (32) ‡ | 120(30) | 150 (32) ‡ | 115 (26) | 146 (32) ‡ | 115 (26) | 142 (31) † | 119 (29) |
| Ventricular volume at timepoint - ml |  |  | 120 (38) ‡ | 94 (21) | 116 (35) ‡ | 89 (22) | 110 (36) † | 88 (24) |
| Reduction of ventricular volume from baseline - ml |  |  | 30 (18) | 21 (12) | 31 (17) | 25 (13) | 32 (16) | 31 (24) |
| Reduction of ventricular volume from baseline - % |  |  | 21 (16) | 18 (8.7) | 22 (14) | 22 (10) | 24 (13) | 25 (14) |
| Intracranial CSF - ml | 425 (66) ‡ | 373 (54) | 404 (78) † | 355 (55) | 405 (72) ‡ | 357 (60) | 419 (74) † | 372 (55) |
| Extraventricular CSF - ml | 276 (51.43) | 253 (44) | 284 (54) | 261 (44) | 290 (57) | 269 (47) | 309 (58) | 283 (40) |
| Ratio ventricular volume/intracranial volume | 0.094 (0.017) | 0.084 (0.017) | 0.075 (0.021) | 0.066 (0.013) | 0.073 (0.020) | 0.063 (0.013) | 0.070 (0.020) | 0.062 (0.016) |
| Ratio ventricular volume/extraventricular CSF | 0.55 (0.15) | 0.49 (0.14) | 0.43 (0.14) | 0.36 (0.085) | 0.41 (0.13) † | 0.34 (0.082) | 0.36 (0.13) | 0.31 (0.078) |
| Ratio ventricular volume/intracranial CSF | 0.35 (0.061) | 0.32 (0.063) | 0.29 (0.070) | 0.26 (0.044) | 0.28 (0.067) | 0.25 (0.044) | 0.26 (0.067) | 0.24 (0.043) |
| Ratio intracranial CSF/intracranial volume | 0.27 (0.033) | 0.26 (0.029) | 0.25 (0.042) | 0.25 (0.032) | 0.26 (0.039) | 0.25 (0.034) | 0.27 (0.038) | 0.26 (0.032) |

**Supplementary Table 3 - Differences in normalized and accessory volumetric measurements between responders and nonresponders at 3, 12 and 36 months**

The values are the means±SDs. † Significant difference from nonresponders at the 0.05 level.

| Timepoint | 3 months | | 12 months | | 36 months | |
| --- | --- | --- | --- | --- | --- | --- |
| Patients assessed - n | 42 | | 43 | | 35 | |
| Outcome | Responders | Nonresponders | Responders | Nonresponders | Responders | Nonresponders |
| Patients n (%) | 36 (86) | 6 (14) | 34 (79) | 9 (21) | 23 (65) | 12 (35) |
| Age | 77 (5.2) | 78 (5.0) | 78 (4.7) | 80 (4.6) | 79 (5.2) † | 83 (3.8) |
| Intracranial CSF - ml | 376 (71) | 407 (72) | 379 (64) | 394 (93) | 385 (54) | 402 (87) |
| Extraventricular CSF - ml | 269 (48) | 298 (61) | 278 (49) | 285 (70) | 292 (43) | 298 (61) |
| Ratio ventricular volume/intracranial volume | 0.071 (0.019) | 0.071 (0.015) | 0.067 (0.017) | 0.071 (0.018) | 0.063 (0.017) | 0.071 (0.019) |
| Ratio intracranial CSF/intracranial volume | 0.25 (0.038) | 0.26 (0.030) | 0.25 (0.034) | 0.26 (0.046) | 0.26 (0.030) | 0.27 (0.041) |
| Ratio ventricular volume/intracranial CSF | 0.28 (0.060) | 0.27 (0.063) | 0.26 (0.060) | 0.27 (0.057) | 0.24(0.060) | 0.26 (0.043) |
| Ratio ventricular volume/extraventricular CSF | 0.40 (0.12) | 0.38(0.12) | 0.37 (0.12) | 0.39 (0.11) | 0.32 (0.11) | 0.35 (0.079) |

**Supplementary figure 1. Gait velocity and changes in gait velocity**

*Left column*: Gait velocity (m/s) at A. Baseline in 50 patients B. 3 months in 41 patients C. 12 months in 43 patients D. 36 months in 35 patients. *Right column:* Change in gait velocity (m/s) compared with baseline at E. 3 months in 41 patients F. 12 months in 43 patients G. 36 months in 35 patients. The overlayed curved line shows the distribution at different timepoints. The vertical red dashed line represents the threshold of meaningful change for gait velocity in older adults of 0.1 m per second.


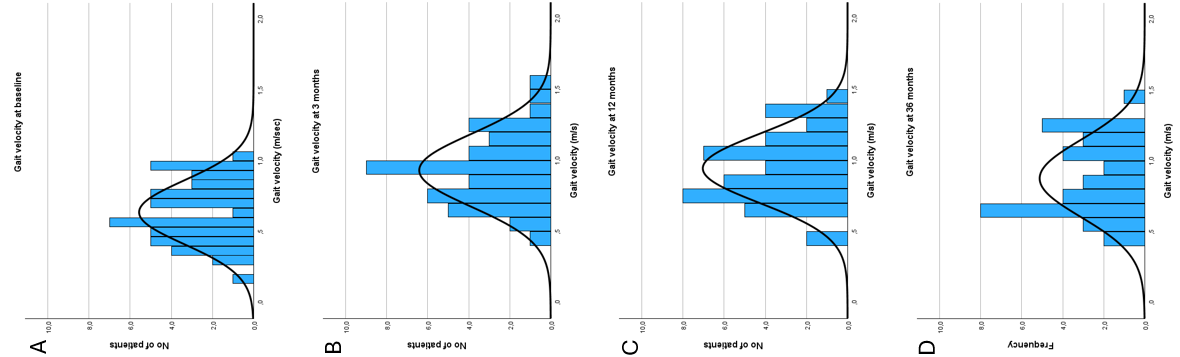

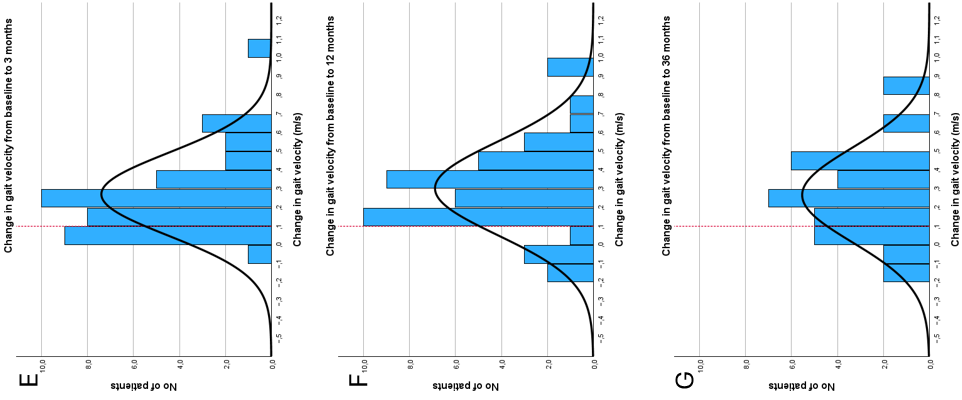


**Supplementary figure 2 - Ventricular volume in patients with unchanged shunt valve setting for 3 years**

The graph shows the mean ventricular volume in 24 patients who completed baseline and at all three follow-up visits with the same shunt setting (Codman Certas Plus without Siphonguard, setting 4 = 110 mm H₂O) for three years. The 3-month time point was significantly different from baseline at the p=0.001 level (‡) and from the 12- and 36-month time points at the p=0.05 level (†).


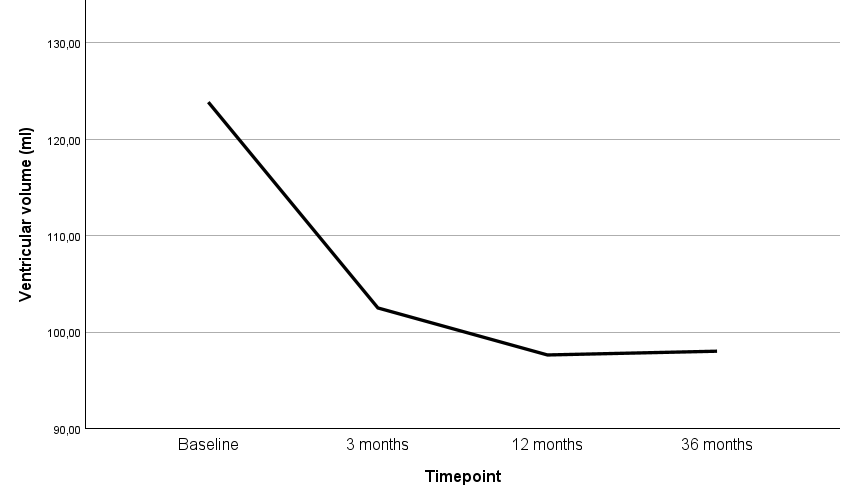


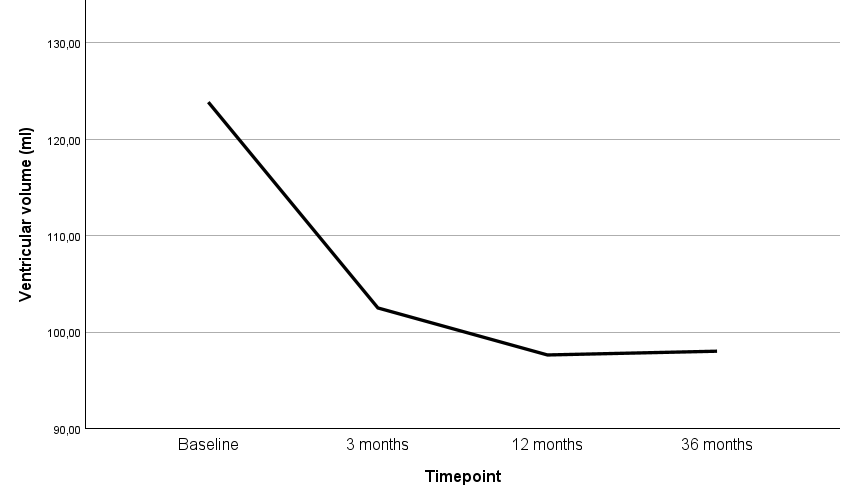


‡

†

**Supplementary figure 3 - Three patients with shunt failure diagnosed by ventricular volumetry**

The diagram shows longitudinal monitoring of ventricular volumes in three patients with shunt failure. Shunt failure was suspected because the ventricular volume at follow-up was greater than or equal to the baseline volume.

**
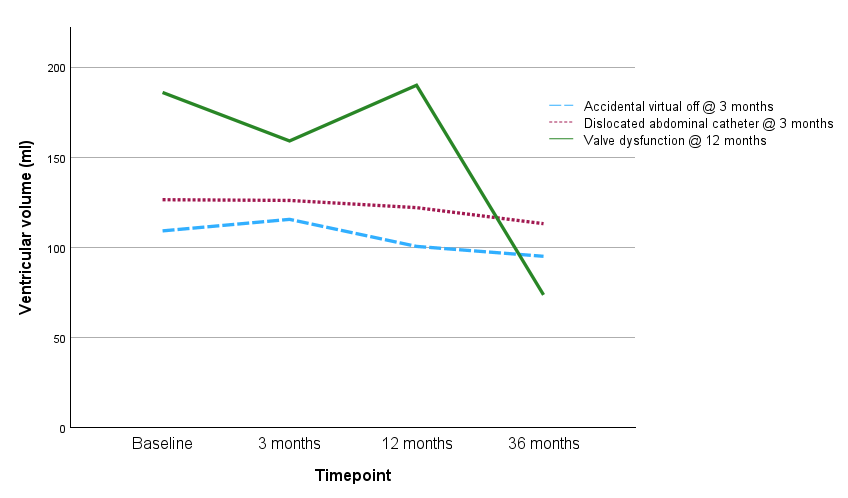
**
